# Accuracy of Rapid Urease Test in Diagnosing *Helicobacter pylori* Infection in Patients using NSAIDs

**DOI:** 10.4103/1319-3767.61238

**Published:** 2010-04

**Authors:** Mojgan Foroutan, Behnam Loloei, Shahrokh Irvani, Ezanollah Azargashb

**Affiliations:** Department of Gastroenterology, Tehran, Iran; 1 Department of Shahid Beheshti University of Medical Sciences, Tehran, Iran; 2 Department of Military University, Tehran, Iran; 3 Department of Epidemiology, Tehran, Iran

**Keywords:** Diagnosis, *Helicobacter pylori*, NSAIDs

## Abstract

**Background/Aim:**

This study aimed to determine the effect of nonsteroidal anti-inflammatory drugs (NSAIDs) on the results of rapid urease test (RUT).

**Patients and Methods:**

The study evaluated 210 consecutive patients for the diagnosis of *Helicobacter pylori (H. pylori)* infection. They were divided into case and control groups based on history of NSAID use (n=70 each). Two biopsy specimens were collected from antrum and corpus of stomach during endoscopy and sent for rapid urease testing and histopathology. Sensitivity, specificity, and accuracy rate of RUT test were compared against histology.

**Results:**

The average age was 55.2±12.9 and 43.3±12.1 years in the case and control groups, respectively. Among NSAID users, RUT sensitivity, specificity and accuracy rate were all 100%. The sensitivity, specificity and accuracy rate of RUT in patients without history of NSAID use were 97.37, 98.57 and 98.14%, respectively. The overall sensitivity, specificity and accuracy rate of RUT were 98.57, 99.29, and 99.04%, respectively.

**Conclusion:**

Our study shows that sensitivity, specificity and accuracy rate of RUT are not affected by NSAID use. Rapid urease test remains a reliable test for diagnosis of *H. pylori* in patients on NSAIDs.

*Helicobacter pylori* (*H. pylori*) is a spiral, gram negative, microaerophilic, urease producing organism that lies in the interface between the gastric epithelial cell surface and overlying mucus gel.

The prevalence of *H. pylori* varies greatly in different parts of the world. It ranges from 8.9 to 72.8% among children from developed and developing countries, respectively, with the re-infection rate also being significantly higher in the latter.[1]

In a recent study in south of Iran, the prevalence rates were 82%, 98%, 88%, 89%, and 57% in age groups of nine months, and two, six, 10, and 15 years, respectively.[2] In another recent study in the north of Iran, prevalence of *H. pylori* infection in a similarly aged population, was reported as 57.8% by histology, rapid urease test (RUT) and serology.[3]

Inadequate sanitation, low socioeconomic status and overcrowding seem to be related to a higher prevalence of *H. pylori* infection. No predominant route of transmission has been identified, and possibilities include the fecal-oral, oro-oral and gastro-oral routes.[4]

Essentially, all *H. pylori* infections lead to chronic gastric inflammation, but this condition is in itself asymptomatic. Symptoms are usually related to illnesses like peptic ulcer disease and gastric adenocarcinoma. The presence of *H. pylori* is also strongly related to gastric lymphoma. Low- grade B-cell mucosa associated lymphoid tissue (MALT) lymphomas which are antigen driven often regress following eradication of *H. pylori*.[5]

Tests for *H. pylori* can be divided into two groups: invasive tests, which require upper gastrointestinal endoscopy and analysis of gastric biopsy specimens, and noninvasive tests. One of the rather widely used invasive tests is the RUT. It is based on the principle that abundant urease enzyme produced by *H. pylori* hydrolyses urea to ammonia. The consequent rise in pH of the medium is detected by phenol red indicator.[6] Several modifications of Christensen's original urea medium have been developed to obtain quick results and improve sensitivity and specificity. Although sensitivity and specificity of RUT are generally above 90%, the test results seem to be influenced by the consumption of several drugs including proton pump inhibitors (PPIs), antibiotics, H2 receptor antagonists, and bismuth.[7] Further, there have been reports of anti *H. pylori* effects of salicylates and sulindac; their effects on the result of RUT are, however, not yet clear.[8]

In the light of the prevalence of *H. pylori* infection, cost of treatment and need for correct diagnosis prior to initiation of eradication treatment, we conducted this study to determine if nonsteroidal anti-inflammatory drugs (NSAID) use adversely influenced RUT results.

## PATIENTS AND METHODS

We conducted a case-control study on patients being referred to the gastroenterology clinic of Imam Hossein Hospital in Tehran, Iran in 2004. All the patients chosen were being evaluated for *H. pylori* infection. Patients who had a history of PPI, H2 receptor antagonist, warfarin, fluoxetin, or steroid use within one week before endoscopy or, antibiotic use within four weeks before endoscopy as well as those with severe medical illness, active gastrointestinal bleeding, and history of gastric surgery and *H. pylori* eradication, were excluded from the study. An informed consent from all patients and approval from the research ethics committee of the faculty of medicine were obtained before proceeding.

Information on age, gender, chief complaint, and type and duration of NSAID use was gathered using a questionnaire. A gastroenterologist performed an upper gastrointestinal (GI) endoscopy using Olympus GIF-100 for all patients and obtained two biopsy specimens from the antrum and two biopsies from the corpus of the stomach. One biopsy from each region was sent for RUT and one for pathologic study using Giemsa staining. The RUT was performed using Chem Enzym Co. kit and read within two hours for all cases. Results of the pathologic studies were considered the gold standard for diagnosis of *H. pylori*.

Subsequently, patients were divided into case and control groups, based on the result of the RUT test and NSAID use. Patients with a negative RUT and a history of NSAID use were assigned to the case group and those with a negative RUT and no history of NSAID use were assigned to the control group. Our study also included a third group which included patients with positive RUT result regardless of history of NSAID use. Pertinent data was recorded in a data sheet and analyzed using SPSS, χ^2^ test.

## RESULTS

A total of 210 patients were studied including 70 with a negative RUT test and a history of consumption of a variety of NSAIDs (case group), 70 with a negative RUT test and no history of NSAID use (control group), and 70 with a positive RUT test. Of the 70 patients with a positive RUT, 38 had no history of NSAID use. The case group included 42 (60%) females and 28 (40%) males. The control group included 37 (52.8%) females and 33 (47.1%) males. The groups lacked any significant gender difference (*P*=0.4). The group with a positive RUT included 41 females (58.7%) and 29 males (41.4%) and was not significantly different from the other two groups in this regard.

The average age was 55.2±12.9 (range: 27 to 82 years) and 43.3±12.1 (20 to 68 years) years in the case and control groups, respectively. In the RUT positive group, the average age was 54.1±11.9 (range: 25 to 71) years.

Among our patients, the most commonly used NSAID was ASA 100 mg (30%) followed by ASA 80 mg (20%), diclofenac (19%), ibuprofen (16%), piroxicam, (7%), indomethacin (6%), naproxen (1%), and ASA 500 mg (1%). Sixty per cent of our patients had used NSAIDs for more than four weeks (mainly ASA 80 and 100 mg) and 22.4% for less than one week. Using pathology results as the gold standard, we calculated sensitivity, specificity and accuracy rates of RUT. Only one false negative case was observed among 140 RUT results and that single case belonged to the control group. Sensitivity, specificity and accuracy rates of RUT were 100% among NSAID users. In patients without any history of NSAID use, sensitivity, specificity and accuracy rate of RUT were 97.4, 98.5 and 98.1%, respectively. The overall sensitivity, specificity and accuracy rate of RUT were 98.6, 99.29, and 99.04%, respectively. The mentioned findings are summarized in Table 1.

**Table 1:** Comparison of characteristics of patients with negative RUT with, and without history of NSAID use

**Group**	**Age (year) (mean±SD)**	**Sex frequency (%)**		**Sensitivity (%)**	**Specificity (%)**	**Accuracy rate (%)**
					
		**Male**	**Female**			
History of NSAID usage	55.2±12.9	28 (40)	42 (60)	100	100	100
No history of NSAID usage	43.3±12.1	33 (52.85)	37 (47.15)	97.37	98.57	98.14

Figures in parenthesis are in percentage

## DISCUSSION

The RUT test is widely used in diagnosis of *H. pylori* infection around the world due to a number of accompanying advantages, including less expense and more rapid results compared to histology or culture. Further, RUT has been shown to have high sensitivity, specificity and clinical accuracy. Said *et al.* reported a sensitivity, specificity, positive predictive value, negative predictive value and diagnostic accuracy of 98, 100, 100, 98 and 99%, respectively for RUT (Pronto Dry).[9]

However, the test has been shown to be less sensitive in case of concurrent use of proton pump inhibitors[10] bismuth and anti-*helicobacter* antibiotics. The test is also influenced by the pH of the gastric mucosa.[11]

Several studies have focused on the effect of medications other than conventional antibiotics on the growth of *H. pylori*. Shirin *et al.* reported that certain NSAIDs, including sodium salicylate, ibuprofen, indomethacin, the selective cyclooxygenase-2 inhibitor NS-398 and two derivatives of sulindac sulfoxide, possess *in vitro* antibacterial activity against *H. pylori* at therapeutically achievable doses.[7] In another *in vitro* study, Wang *et al.* demonstrated that Aspirin inhibited the growth of *H. pylori*, suppressed the mutagenic effect of metronidazole, and enhanced the susceptibility of *H. pylori* to antimicrobial agents.[12]

These studies have focused on the *in vitro* effect of NSAIDs on the growth of *H. pylori* and one cannot extrapolate the results to *in vivo* settings and test results since organisms are multiplied in the urea broth during the test.

This is supported by the work of Graham and colleagues who reported that *H. pylori* was not susceptible to aspirin and other NSAIDs, such as indomethacin, ibuprofen, naproxen, or tolmetin *in vivo*.[13] Similarly, our results showed that RUT maintains its high sensitivity and specificity even in patients who have used NSAID for a moderate period of time. One of the few studies in this regard is that performed by Caselli *et al.* in 1989. Caselli *et al.* reported that Campylobacter-like organisms were detected in a significantly lower proportion (*P*<0.001) of rheumatoid arthritis (RA) patients than outpatients (30.6 and 59.0%, respectively).[14] However, this study was greatly limited in that the authors used only one method for the diagnosis of *H. pylori,* namely biopsy, and it is reasonably possible that the organisms did not exist in the exact region where the biopsy was taken. The mentioned study is also limited by the selective nature of its sample i.e. RA patients who frequently use a wide range of other medications, that could interfere with test results. Moreover, other studies have demonstrated that there is no meaningful difference in *H. pylori* prevalence between NSAID users and non-users.[15,16] The relatively small sample size and absence of long-term NSAID users in our study warrants further research on the subject.

## CONCLUSION

Our study results show that NSAID use does not give false negative results in RUT; hence it is not necessary to stop NSAIDs before RUT.
